# Bajiquan martial arts training as physical activity for enhancing physical fitness, body composition, and perceived exercise benefits: a quasi-experimental study

**DOI:** 10.3389/fspor.2025.1545481

**Published:** 2025-03-31

**Authors:** Chi-Te Wang, Cheng-Wen Tien, Wen-Ching Huang

**Affiliations:** ^1^ Department of Exercise and Health Science, National Taipei University of Nursing and Health Sciences, Taipei, Taiwan; ^2^ General Education Center, National Taipei University of Nursing and Health Sciences, Taipei, Taiwan

**Keywords:** traditional martial arts, physical fitness, recreation activity, health promotion, Bajiquan

## Abstract

**Background:**

Martial arts are a traditional aspect of Chinese culture, and with the diverse development of recreational activities, they have gained widespread acceptance not only for self-defense but also as a popular recreational activity. Physical activity and fitness characteristics associated with different martial arts vary depending on developmental backgrounds. Bajiquan is a traditional Chinese martial art known for its explosive power in close combat, emphasizing quick elbow and shoulder strikes over a short range. However, research on the application of Bajiquan to physical activity, health promotion, and its perceived benefits remains relatively limited. This study aimed to investigate the effects of a programmed 8-week Bajiquan training intervention on physical fitness, body composition, and perceived benefits of exercise.

**Methods:**

This quasi-experimental study enrolled participants and allocated them to the experimental (*n* = 15; 27.4 ± 2.6 years; female = 13.3%) and control groups (*n* = 15; 26.0 ± 3.1 years; female = 13.3%). The experimental group participated in an 8-week Bajiquan program, whereas the control group engaged in regular exercise with the same intervention frequency. Pre- and post-tests were conducted to assess the effects on physical fitness and body composition. Additionally, participants' subjective perceptions of the benefits of martial arts exercise were evaluated using an exercise perceived benefit questionnaire.

**Results:**

The experimental group demonstrated significantly higher social relationships (Δ = 17.2%; δ = 0.586, *p* < 0.05) and personal benefits (Δ = 19.8%; δ = 0.431, *p* < 0.05) than the control group (*p* < 0.05). Changes in pre- and post-test measurements within the experimental group were significantly different from those in the control group in terms of body weight (*p* = 0.008, *d* = 1.05), body mass index (*p* = 0.003, *d* = 1.17), and body fat percentage (*p* = 0.004, *d* = 1.13). The experimental group exhibited significant differences in changes in muscle explosive power (*p* = 0.003, *d* = 1.27), cardiorespiratory fitness (*p* = 0.004, *d* = 1.14), and core muscle strength (*p* = 0.009, *d* = 1.10) compared with the control group. Core muscle strength also significantly increased in the experimental group compared to that in the control group in the post-test (Δ = 17.0%; *p* = 0.003, *d* = 1.21).

**Conclusions:**

The Bajiquan martial arts exercise intervention demonstrated beneficial effects on physical and mental development, making it a viable option for physical activity programs. In the future, program adjustments and applications can be tailored for different populations, such as children or the elderly, to promote health and disseminate the practice of martial arts.

## 1 Introduction

Bajiquan is a traditional Chinese martial art originating in Hebei Province, northern China. It is characterized by explosive power and short-range force, with a focus on elbow strikes and close-quarter combat. Bajiquan has gained popularity in recent decades in northern China and Taiwan due to its self-defense applications and unique martial arts characteristics. It has since expanded to Japan, South Korea, and various Western countries. The essence of Bajiquan lies in the application of explosive power, which is achieved through instantaneous acceleration from the waist to the limbs during attacks. Its primary techniques include elbow strikes, arm punches, fist strikes, hip strikes, and shoulder strikes, with a strong emphasis on lower-body training and footwork coordination (1). Martial arts training, which emphasizes full-body movement patterns, philosophy, interpersonal interactions, and self-defense, positively affects physical activity, mental health, and cognitive well-being in adults and children. Martial arts are a form of exercise therapy that can be provided to people of all ages to promote holistic health (2). In the elderly population, Judo practice may have positive effects on three main areas: physiological health (including bone health, anthropometric measures, and quality of life), functional fitness (such as balance, strength, and walking speed), and psychosocial well-being (including fear of falling, cognitive function, and self-efficacy) (3). In recent years, the number of people participating in martial arts has increased steadily. A report on martial arts participation in Australia indicates that 1.2% of the adult 15+ population engages in martial arts, with 85% of participation being organized. The total annual spending on martial arts reaches $87 million. Additionally, there is a 49% potential market growth, with strong interest across all age groups, particularly among older adults (65+) considering participation (4). This indicates a growing trend in using martial arts as a form of exercise or physical activity.

The application of martial arts in health promotion is particularly beneficial for older adults as it can improve strength, mobility, aerobic endurance, flexibility, and balance (5). For adults, the positive effects of martial arts practice include improvements in balance, cognitive function, and mental health. However, further investigation and long-term follow-up are required to fully understand these benefits (6). Previous studies on young adults have also examined the modulation of hormones, such as oxytocin, in relation to martial arts training, highlighting that its effects may vary across different populations. Additionally, martial arts experience has been associated with enhanced selective attention (7, 8). Evidence also demonstrates a positive correlation between practicing martial arts and health behaviors and higher scores in quality of life (9). These studies demonstrated that martial arts can be used as a form of physical activity and therapeutic intervention, offering health and developmental benefits across different populations.

Globally, one-third of the age-standardized adult population (31.3%) is physically inactive, as indicated by population-based surveys (10). Sedentary behavior has numerous detrimental effects on health including increased risks of all-cause mortality, cardiovascular disease mortality, cancer, metabolic disorders, mental health issues, and cognitive impairment. Reducing sedentary behaviors and increasing physical activity are crucial for promoting public health (11). Increasing physical activity is effective in preventing chronic diseases and premature mortality, with a positive relationship observed between physical activity and health outcomes (12). In addition to improving obesity and physical fitness, increased physical activity plays a vital role in emotional stability and mental health (13). Previous research has shown that martial arts such as Taekwondo positively influences body composition and reduces obesity (14). In addition, findings from the literature review indicate that Judo positively impacts children and adolescents by improving body fat composition, modulating bone mineralization, and enhancing the cardiorespiratory system (15). However, owing to the limitations of existing studies, such as a lack of research, heterogeneity, short intervention periods, small sample sizes, and methodological constraints, whether martial arts are effective in improving anthropometric measures and body composition in individuals with overweight and obesity remains unclear (16). Therefore, whether martial arts can be considered suitable for weight control requires further evaluation.

The impact of martial arts on health has been studied primarily in specific disciplines. Tai Chi is the most extensively studied form of martial arts, followed by Judo, Karate, and Taekwondo. Although further standardized research is needed, existing evidence suggests that traditional martial arts can serve as exercise prescriptions for various populations (17). In recent years, martial arts research has primarily focused on mixed martial arts, such as Tai Chi, Judo, Karate, and Taekwondo. Martial arts emphasize not only self-defense but also movement patterns and interpersonal interactions. They can be considered a form of exercise therapy that benefits physical activity, psychological well-being, and cognitive health across all age groups (2, 6). However, empirical research on Bajiquan is lacking. Therefore, this study aims to examine the effects of a Bajiquan training program on body composition and physical fitness. Additionally, the perceived benefits of martial arts will be assessed through the development of a Martial Arts Perceived Benefits Questionnaire, ensuring validated reliability and validity. The findings of this study hold significant implications for the future application and promotion of Bajiquan as a means to achieve health promotion objectives.

## 2 Materials and methods

### 2.1 Experimental design

Participants were recruited from fitness centers and screened based on the following eligibility criteria. The study groups (experimental and control) were selected using convenience sampling for an exploratory study in present study. The experiment was based on quasi-experimental design with a parallel control group for pretest–posttest evaluations following the martial intervention (duration: 2023.4.3 to 2023.7.3). The inclusion criteria were as follows: no experience of any musculoskeletal injuries within the past month and ability to engage in normal training activities; without cognitive impairment; capability to understand the questionnaire; and ability to complete it online. The exclusion criteria were as follows: diagnosis of musculoskeletal disorders, joint diseases, unstable cardiovascular conditions, or cognitive impairments; deemed unfit for physical activity by a physician; and inability to understand or complete the questionnaire online. All participants provided written informed consent. This study implemented an 8-week Bajiquan Martial Arts Training Program as an intervention. Pre- and post-intervention assessments were conducted to evaluate the effects of martial arts training on body composition, functional fitness, and the perceived benefits of exercise. This study was reviewed and approved by the Fu Jen Catholic University Institutional Review Board (New Taipei City, Taiwan; C111137) (valid duration: 2023.3.29 to 2024.3.28).

### 2.2 Bajiquan martial arts training program

Certified martial arts instructors led the experimental group in Bajiquan training. The instructor possessed the following certifications: Bajiquan Instructor Certification from Wu Tan Bajiquan, Coaching Certification from the Chinese Wushu Sanda Association, and Level C Martial Arts Coach Certification from the Taipei City Sports Federation. The training followed a systematic and progressive approach to physical and physiological adaptations. The course began with fundamental training in stance work, hand form, and footwork. This was followed by practicing five forms of routine, starting with “Xiao Baji” to establish the foundation, “Da Baji” to advance techniques, and “Liu Da Kai” to master specific skills. “Lian Huan Quan”, created by Master Liu Yunqiao, was a comprehensive adaptation of the first three forms. Finally, participants practiced “Pigua Zhang”, focusing on long-range strikes and balancing weaknesses and strengths. Each section of the curriculum was taught for 10–12 h per week, with an additional 12 h for comprehensive review, totaling 84 h. Training sessions were implemented 5–6 times per week, with each session lasting 2 h, for a total duration of 8 weeks (Table 1). The control group participated in general fitness training including swimming, yoga, running, and fitness exercises. Specific examples of the training included 50-meter freestyle or breaststroke swimming, 3,000-meter running, beginner-level yoga, and basic fitness routines. The overall exercise duration and frequency were the same as those of the experimental group.

**Table 1:** Bajiquan martial arts training program.

Weeks	Training topics	Hours	Frequency
1st	fundamental training^a^	10	5 times/week
2ed	fundamental training^a^	2	5 times/week
	Xiao Baji	8	
3ed	Xiao Baji	4	5 times/week
	Da Baji	6	
4th	Da Baji	6	6 times/week
	Liu Da Kai	6	
5th	Liu Da Kai	6	6 times/week
	Lian Huan Quan	6	
6th	Lian Huan Quan	6	5 times/week
	Pigua Zhang	4	
7th	Pigua Zhang	8	5 times/week
	General review	2	
8th	General review	10	5 times/week
Bajiquan demonstration video (Da Baji, Lian Huan Quan)^b^: https://www.youtube.com/watch?v=C4xblWK6e9Y&t=49s			

^a^ Fundamental training includes six basic movements (arm raise and circle, arm swing and waist twist, arm lift and back stretch, left and right palm press, left and right leg thrust, and horse stance and bow punch).

^b^ Martial art was demonstrated by the author.

#### 2.2.1 The forms of routine in Bajiquan

These four forms represent essential routines in present Bajiquan program, each emphasizing distinct techniques and applications. A brief overview is presented as follows.

##### 2.2.1.1 Xiao Baji

Characteristics: An introductory routine featuring compact and powerful movements that focus on fundamental skills and force emission.

Techniques: Emphasizes close-range strikes, explosive short-power (peng jin), elbow strikes (ding xin zhou), and a stable, grounded stance.

Application: Develops footwork stability, enhances close-combat abilities, and establishes the foundation for force generation in Bajiquan.

##### 2.2.1.2 Da Baji

Characteristics: Involves broader movements and a wider attack range, incorporating the full range of Bajiquan techniques.

Techniques: Introduces long-range offensive methods such as lateral palm strikes (cheng zhang), upward elbow strikes (tiao da ding zhou), and continuous kicking techniques.

Application: Enhances full-body coordination and explosive power while balancing offense and defense in combat scenarios.

##### 2.2.1.3 Liu Da Kai

Characteristics: The core combat techniques of Bajiquan, emphasizing breaking through an opponent's defense.

Techniques: Incorporates six primary offensive methods—body collision (tie shan kao), spiraling punch (chan si beng chui), aggressive upward strike (meng hu ying pa shan), lateral palm strikes (zuo you ta zhang), blocking punches (zuo you lan chui), and continuous tiger pounce punches (hu pu lian huan quan).

Application: Designed for breaking an opponent's guard and swiftly engaging in close combat, making it highly applicable in practical combat and Sanda (Chinese kickboxing).

##### 2.2.1.4 Lian Huan Quan

Characteristics: Focuses on seamless, relentless attacks with constant forward pressure.

Techniques: Features uninterrupted punches, elbow strikes, and shoulder thrusts, emphasizing rhythm and aggression.

Application: Trains the ability to sustain consecutive attacks, preventing the opponent from regaining control—an embodiment of Bajiquan's offensive nature.

These forms complement one another, progressing from foundational skills (Xiao Baji) to a complete technical framework (Da Baji), and further into advanced combat applications (Liu Da Kai and Lian Huan Quan). Bajiquan is characterized by close-range combat, explosive short-power, and dynamic force application, and each of these routines is structured around these core principles.

### 2.3 Body composition

Body composition was measured using four-point bioelectrical impedance of the hands and feet. This method reduces measurement errors caused by uneven body posture or fluid distribution between the upper and lower bodies, providing a more accurate assessment of whole-body composition. Body composition including body fat percentage (BFP), visceral fat (VF), and muscle mass was measured before and after the intervention using portable bioelectrical impedance analysis devices (InBody H20B; Seoul, South Korea).

### 2.4 Functional fitness tests

Cardiovascular endurance, explosive power, and core strength endurance were evaluated using a 3 min step, horizontal jumps, and bent-knee sit-up tests, respectively.

#### 2.4.1 Cardiovascular endurance

The step test was used to assess heart rate recovery over a three-minute period following a standardized stepping exercise. Participants stepped onto a 35 cm-high wooden crate at a consistent pace of 24 cycles per minute for three minutes, with each cycle consisting of two steps up and two steps down, synchronized to a metronome set at 96 beats per minute. A stopwatch recorded the actual exercise duration. Regardless of whether participants completed the full three-minute exercise or stopped prematurely, their heart rates were measured while seated at three intervals: 1:00–1:30 min, 2:00–2:30 min, and 3:00–3:30 min post-exercise. The following formula was used to calculate the stepping cardiovascular function index: (exercise duration × 100)/(2 × sum of the three pulse rates) (18).

#### 2.4.2 Explosive leg power

Lower body explosive strength was evaluated through standing long jump and standing triple jump tests, which are widely used to assess leg power. In the standing long jump, participants positioned their feet slightly apart behind a marked line on a calibrated landing mat. They executed a simultaneous two-foot takeoff and landing, using arm swings and knee flexion to maximize jump distance. The distance from the starting line to the nearest landing point (heel position) was recorded and rounded down to the nearest centimeter for analysis (18).

#### 2.4.3 Muscular endurance

Muscular endurance was assessed using the 1 min bent-knee sit-up test. Participants lay on a mat with their arms crossed over their chests and hands resting lightly on their shoulders. Each sit-up required them to curl up until both elbows touched their knees, then return to the starting position with both shoulder blades touching the mat before initiating the next repetition. The total number of correctly performed sit-ups within 60 s was recorded as a measure of core endurance. The detailed procedures, devices, and manipulations have been described previously (18).

### 2.5 Martial arts exercise perceived benefits questionnaire

In this study, reliability and validity of the questionnaire were analyzed. A pilot survey was conducted online, which yielded 449 valid responses. The original questionnaire contained 30 items; however, two items were excluded because of inadequate decision values and correlation coefficients. After applying factor analysis using the orthogonal rotation method with maximum variance, items 13 and 27 were removed as the fifth factor contained only these two items. Thus, a total of 26 items were retained. A 5-point Likert scale was used for the questionnaire. The reliability analysis of the questionnaire yielded a Cronbach's α coefficient of 0.938, indicating high internal consistency. The Kaiser–Meyer–Olkin value for the factor analysis was 0.943, and Bartlett's test of sphericity produced a significant chi-square value (*p* < 0.05), confirming the suitability of the data for the factor analysis (Table 2). Factor analysis divided the questionnaire into four dimensions as follows: “personal”, “social”, “psychological”, and “physiological barriers to exercise”. The procedures of reliability, validity, and factor analysis for this questionnaire were provided as complementary files for reference.

**Table 2:** The factor analysis for martial arts exercise perceived benefit questionnaire.

Factor analysis/ dimensions	Personal benefits	Social relationship benefits	Psychological barriers	Barriers to exercise
Eigenvalues	6.05	5.05	3.30	2.62
Retained question number	11	7	4	4
% of variance	23.26%	19.41%	12.68%	10.10%
Cumulative %	65.44%			
Individual Cronbach's α	.940	.893	.854	.819
Overall Cronbach's α	.938			
KMO	.943			
Bartlett's test	*χ*^2^ (325, *N* = 449) = 7587.56, *p* < .001			

KMO, Kaiser–Meyer–Olkin.

### 2.6 Power analysis

The repeated measures design equation implemented in G*Power software (version 3.1.9.6) was used to calculate the required statistical power of 80%. The sample size estimation was based on an effect size of 0.4, a Type I error probability of 0.05, and the *t*-test family. The analysis determined that a total of 50 participants would be necessary to proceed with the intervention. While the present study had limited statistical power to detect significant effects, it was expected to be sufficient for identifying trends in improvements, which could be further explored in a larger follow-up study.

### 2.7 Statistical analysis

The anthropometric and sociodemographic characteristics of the participants were analyzed using descriptive statistics and a chi-squared test. The normality of all dependent variables was assessed using the Kolmogorov–Smirnov test to determine the appropriateness of parametric and non-parametric analyses. Non-parametric tests, including the Wilcoxon signed-rank test and the Mann–Whitney *U*-test, were employed for the analysis of questionnaire assessments. In contrast, parametric methods, such as mixed two-way analysis of variance (ANOVA) and unpaired and paired *t*-tests, were utilized to evaluate body composition, physical fitness, and baseline anthropometric variables. The gain score (delta value) and effect sizes (ES), such like Cohen's d or Cliff's δ, were calculated for all significant findings. These statistical methods were applied to identify significant differences in the dependent variables both between and within groups. SPSS version 22 (IBM, Armonk, NY, USA) was used for all the statistical analyses. The probability of a type I error (*p* < 0.05) was considered significant.

## 3 Results

### 3.1 Anthropometric and sociodemographic characteristics of participants

In the Table 3, no significant differences in age or weight were observed between the two groups, although the participants in the control group were significantly taller than those in the experimental group. The chi-square tests showed no significant differences in categorical variables such as sex, marital status, education level, and occupation between the groups (Table 3).

**Table 3:** Baseline anthropometric and sociodemographic characteristics of participants.

Characteristics	Experimental group (*N* = 15)	Control group (*N* = 15)
Sex (male/female)	13/2	13/2
Age (years)	27.4 ± 2.61	26.0 ± 3.09
Height (cm)	170.13 ± 5.71	176.87 ± 7.28
Weight (kg)	72.7 ± 11	74.1 ± 11
BMI	25.0 ± 2.9	23.4 ± 2.2
Marital status^+^		
Married	12	12
Single	3	3
Academic qualification^+^		
Senior high school	4	3
College	10	10
Graduate school	1	2
Occupation^+^		
Student	10	13
Public office	3	2
Freelance	2	0
Regular exercise^+^		
None	0	0
3 or less times/week	12	11
4 or more times/week	3	4

^+^ Was indicated as number distribution.

### 3.2 Effects of Bajiquan martial arts training on exercise perceived benefits

The martial arts exercise perceived benefit questionnaire encompasses four dimensions: personal, social, psychological, and physiological barriers to exercising. This scale demonstrated effective reliability and validity in evaluating the psychological effects of Bajiquan training (Table 4). Among the personal benefits, improvements in physical fitness, coordination, explosiveness, and lower body strength were ranked as the most significant. Following the intervention, improvements in physical fitness (Cliff's δ = 0.484) and explosiveness (Cliff's δ = 0.466) as well as in the overall personal benefit dimension (Cliff's δ = 0.431) were significantly higher in the experimental group than in the control group. In addition, motivation (Cliff's δ = 0.435), shared experiences (Cliff's δ = 0.537), and learning different perspectives (Cliff's δ = 0.480), along with the overall Social Relationship Benefits dimension (Cliff's δ = 0.586), showed significant improvements compared to the control group. In the barrier dimension, perceived pressure during martial arts sessions (Cliff's δ = 0.484) demonstrated a significant decrease following the implementation of martial arts training.

**Table 4:** Effects of Bajiquan martial arts training on perceived exercise benefits.

Dimension	Items	Experimental group		Control group	
		Pre-test	Post-test	Pre-test	Post-test
Personal benefits	Significant improvement in physical fitness	4.00 ± 0.76	4.27 ± 0.80^a^	3.6 ± 0.7	3.33 ± 1.1
	Better body coordination	4.20 ± 0.86	4.27 ± 0.70	3.5 ± 0.9	3.53 ± 1.1
	Sufficient explosiveness	4.07 ± 0.88	4.07 ± 0.88^a^	3.5 ± 0.8	3.33 ± 0.8
	Strong lower body strength	4.27 ± 0.88	4.33 ± 0.72	3.9 ± 1.2	3.53 ± 1.2
	Improvement in flexibility	4.13 ± 0.74	3.93 ± 0.96	3.7 ± 1.1	3.53 ± 1.2
	Developed a regular martial art practice routine	3.73 ± 0.88	3.67 ± 0.82	3.3 ± 0.9	3.00 ± 1.3
	Reduced work stress	3.73 ± 1.10	3.47 ± 0.92	3.2 ± 0.9	2.87 ± 1.3
	Feeling happy	3.67 ± 1.05	3.60 ± 0.99	3.1 ± 1.1	3.00 ± 1.0
	Inner calmness	3.73 ± 0.88	3.67 ± 0.90	3.3 ± 1.1	3.27 ± 1.1
	Increased focus	3.93 ± 0.96	3.87 ± 0.83	3.4 ± 0.8	3.27 ± 1.2
	More self-confidence	3.93 ± 0.88^a^	3.80 ± 0.94	3.1 ± 1.0	3.13 ± 1.1
	Dimension sum	43.4 ± 8.2	42.9 ± 7.4^a^	37.8 ± 8.6	35.8 ± 10.7
Social relationship benefits	Made like-minded friends	3.73 ± 1.03	3.87 ± 0.99	3.27 ± 1.03	3.13 ± 1.13
	Strengthened friendships	3.80 ± 1.01	3.87 ± 0.83	3.20 ± 1.21	3.27 ± 0.96
	Martial art cannot help others	2.60 ± 1.55	2.53 ± 0.99	2.87 ± 1.13	3.13 ± 1.06
	Motivations to each other	4.13 ± 0.99	4.13 ± 0.74^a^	3.33 ± 1.29	3.33 ± 1.05
	Sharing martial art experiences together	4.07 ± 0.96	4.27 ± 0.70^a^	3.60 ± 1.06	3.27 ± 1.10
	Strengthened family bonds	3.27 ± 0.96	3.20 ± 0.41	2.47 ± 1.25	2.67 ± 1.18
	Learning different perspectives and ideas	4.13 ± 0.83	4.07 ± 0.80^a^	3.60 ± 1.06	3.27 ± 0.88
	Dimension sum	25.7 ± 5.01	25.9 ± 2.91^a^	22.3 ± 4.53	22.1 ± 5.16
Psychological barriers	Feel pressure during each martial art session	2.60 ± 1.18	2.20 ± 0.94^a^	2.93 ± 1.49	3.27 ± 1.16
	Afraid of making mistakes while practicing	3.27 ± 1.22	2.87 ± 1.19	3.07 ± 1.49	3.20 ± 1.26
	Feeling like still not improving	2.33 ± 1.05	2.27 ± 1.03	2.73 ± 1.44	2.87 ± 1.30
	Always struggle to keep up with others	2.67 ± 1.11	1.87 ± 0.92	2.67 ± 1.45	2.73 ± 1.44
	Dimension sum	10.9 ± 3.48	9.20 ± 3.21	11.4 ± 5.50	12.1 ± 4.86
Barriers to exercise	Feeling physically fatigued	3.13 ± 0.99	2.20 ± 1.01	2.67 ± 1.29	2.67 ± 1.29
	Frequent discomfort in joints	3.40 ± 1.18	3.33 ± 1.23	3.33 ± 1.29	3.13 ± 1.30
	Unable to execute movements precisely	2.73 ± 0.88	2.13 ± 0.99	3.07 ± 1.44	3.00 ± 1.31
	Feeling stiff and unable to relax	2.87 ± 0.92	2.27 ± 1.10	3.13 ± 1.46	3.07 ± 1.39
	Dimension sum	12.1 ± 3.41	9.93 ± 3.43	12.2 ± 4.63	11.9 ± 4.47

^a^ Significant differences between groups.

### 3.3 Effects of Bajiquan martial arts training on body composition

The body composition of the participants was assessed before and after the intervention, which involved martial arts and general fitness training. The interaction between treatment and time demonstrated significant differences in body weight, body mass index (BMI), and BFP [F(1, 28) = 8.271, 10.44, and 9.686, *p* < 0.05, *η*p^2^ = 0.228, 0.272, and 0.257, respectively]. Within the experimental group, significant differences in BMI [t(14) = 3.872, *p* = 0.002, *d* = 0.99], BFP [t(14) = 3.832, *p* = 0.002, *d* = 0.98], and VF [t(14) = 2.646, *p* = 0.019, *d* = 0.68] were observed. Meanwhile, no significant differences were observed in the control group. A difference-in-differences analysis, based on gain scores in body weight [t(28) = 2.876, *p* = 0.008, *d* = 1.05], BMI [t(28) = 3.231, *p* = 0.003, *d* = 1.17], and BFP [t(28) = 3.112, *p* = 0.004, *d* = 1.13], revealed that participants in the Bajiquan training group experienced significantly greater improvements than those in the control group (Table 5).

**Table 5:** Effects of Bajiquan martial arts training on body composition and physical fitness.

Items	Experimental group			Control group			*p* value		
	Pre-test	Post-test	△	Pre-test	Post-test	△	Treatment	Time	Interaction
Body weight (kg)	72.7 ± 11	70.5 ± 9.8	−2.2 ± 2.2^#^	74.1 ± 11	73.8 ± 10	−0.27 ± 1.4	0.542	0.001	0.008
BMI (kg/m^2^)	25.0 ± 2.9	24.2 ± 2.7*	−0.7 ± 0.01^#^	23.4 ± 2.2	23.4 ± 2.1	−0.04 ± 0.4	0.219	0.001	0.003
Body fat percentage (%)	24.5 ± 6.5	22.4 ± 5.8*	−2.1 ± 2.1^#^	20.0 ± 7.0	20.0 ± 7.1	0 ± 1.54	0.160	0.004	0.004
Muscular mass (kg)	30.5 ± 4.3	30.3 ± 4.1	−0. 2 ± 0.53	33.5 ± 6.1	33.4 ± 6.1	−0. 1 ± 0.68	0.116	0.277	0.573
Visceral fat (%)	6.9 ± 2.9	6.3 ± 2.3*	−0. 7 ± 0.97	5.7 ± 2.6	5.6 ± 2.7	−0. 1 ± 0.92	0.332	0.028	0.134
3 min stepping	70.8 ± 9.5	80.7 ± 11.5*	9.9 ± 9.1^#^	78.3 ± 14.9	75.2 ± 15.6	−3.0 ± 13	0.822	0.109	0.004
Horizontal jump (cm)	187.5 ± 30.1	206.1 ± 20.9*	18.5 ± 20^#^	190.5 ± 29.0	190.3 ± 29.7	−0. 2 ± 3.9	0.514	0.002	0.002
Bent-knee sit-up (times)	37.3 ± 9.3	46.4 ± 6.3*^,#^	9.1 ± 11^#^	37.7 ± 6.7	38.5 ± 6.8*	0. 8 ± 1.3	0.118	0.001	0.005

△Change (gain scores) within groups. *Significant differences within group. ^#^Significant differences between groups. BMI, body mass index.

### 3.4 Effects of Bajiquan martial arts training on functional fitness

Cardiovascular endurance, explosive power, and core strength endurance were evaluated using the 3 min stepping, horizontal jump, and bent-knee sit-up tests, respectively. The interaction between treatment and time demonstrated significant differences in the 3 min step, horizontal jump, and bent-knee sit-up [F(1, 28) = 9.761, 12.189, and 9.098, *p* < 0.05, *η*p^2^ = 0.259, 0.303, and 0.245, respectively]. The 3 min step (*p* = 0.002, *d* = 0.98), horizontal jump (*p* = 0.003, *d* = 0.91), and bent-knee sit-up (*p* = 0.005, *d* = 0.86) showed significant improvement within the experimental group, and regular fitness training could only significantly elevate core strength endurance (*p* = 0.028, *d* = 0.66) within the control group. In addition, a significant difference in core strength endurance was observed between the two groups (*p* = 0.003, *d* = 1.21) in the post-test. A difference-in-differences analysis, based on gain scores in 3 min stepping (*p* = 0.004, *d* = 1.14), horizontal jump (*p* = 0.003, *d* = 1.27), and bent-knee sit-up (*p* = 0.009, *d* = 1.10), revealed that participants in the Bajiquan martial arts training group experienced significantly greater improvements than those in the control group (Table 5).

## 4 Discussion

Martial arts, which originate from diverse cultures and historical backgrounds, are rooted in various forms of physical activity and fitness. Traditionally considered a method of self-defense and military training, martial arts encompass a broader purpose that includes continuous mental, physical, emotional, and spiritual self-improvement. In recent years, martial arts training has emphasized whole-body movement patterns, philosophical teaching, interpersonal interactions, and functional self-defense. Thus, martial arts have been proposed as a form of exercise therapy suitable for individuals of all ages with the potential to promote health (2. Sun, 2024). However, previous research on martial arts, particularly Bajiquan, remains limited, and further empirical investigation is required to evaluate its potential benefits on functional fitness, as well as its physiological and psychological effects. In the present study, the 8-week Bajiquan Martial Arts Training Program resulted in significant improvements in body composition and physical fitness. Additionally, the martial arts exercise perceived benefit questionnaire effectively assessed improvements in personal and social relationships.

Improvements in physical fitness with various martial arts interventions have been well documented. Tai Chi, one of the most well-known soft martial arts exercises, is an effective neuromotor exercise that emphasizes movement control and sustained stretching across multiple muscle groups. A systematic review has demonstrated that Tai Chi significantly enhances balance, BMI, BFP, vital capacity, and flexibility (as measured by the sit-and-reach test) in adults, which is supported by high-quality evidence. However, its effects on blood pressure regulation and sympathetic activity were insignificant (19). Additionally, Tai Chi significantly improves handgrip strength, 6 min walking distance, standing time in a single-leg stance with open eyes, and thoracolumbar spine flexibility, although further validation is needed for younger, healthy populations (20). Hard martial arts, such as Karate and Taekwondo, are characterized by their emphasis on forceful and direct techniques, relying on strength and aggression. These disciplines often prioritize physical conditioning, powerful strikes, and linear movements. A systematic review of Taekwondo training has revealed significant improvements in cardiopulmonary and muscular endurance among Korean elementary students (21). Additionally, the implementation of Taekwondo has been associated with statistically significant improvements in weight, BMI, waist circumference, waist-to-hip ratio, body fat mass, BFP, lean mass, and muscle mass (14). Consequently, improvements in specific physical fitness and body composition may vary between soft and hard martial arts. Nonetheless, Bajiquan interventions have also demonstrated significant improvements in body weight, BMI, BFP, and aerobic endurance, showing greater effectiveness than other forms of martial arts.

Martial arts training is effective in enhancing physical fitness; however, the extent of these improvements can vary depending on the specific type of martial arts practiced. A previous study comparing the effects of Taekwondo and Wushu on male adolescents has reported that the Wushu group demonstrated significantly higher explosive leg power and superior Wingate anaerobic capacity parameters including mean power, anaerobic capacity, and anaerobic power than the Taekwondo group (22). By contrast, Bajiquan requires a distinct set of physical fitness attributes, with a greater emphasis on explosiveness, kinetic chain strength, and footwork, commonly referred to as Fa Jin (the expression of explosive power), and the study also showed the significant elevation in explosiveness with Bajiquan training. Core training is a novel approach for strength training. Strong core muscles act as the central hub of the kinetic chain, providing a pivotal point for generating limb strength and facilitating the connection and transfer of power between the upper and lower limbs. Core training enhances the transfer of movement and force to distal parts of the body and improves the overall control of physical activities (23). Core training plays an important role in enhancing the functional performance in martial arts. The significance of core strength is often overlooked in martial arts training. However, previous studies have indicated that core training improves functional movement performance in martial arts, particularly in Greco-Roman wrestling, as demonstrated by exercises, such as overhead medicine ball throws, suplexes, bridges, and medicine ball chest throw (24). The significance of core strength training in the martial art Muay Thai could enhance both velocity and force in distal limbs (25). The core strength and explosive power of Bajiquan martial arts training can also significantly improve the speed and power of punches and kicks, demonstrating the effectiveness of enhanced core activation. Improved core strength and explosiveness were significantly observed with Bajiquan martial implementation in the current study. In addition to traditional training methods, further research is required to determine whether practicing various martial arts disciplines can complement and enhance functional fitness and athletic performance.

Previous studies have indicated that the intensity and mode of swimming exercises lead to different adaptations in physical fitness. High-intensity interval swimming has been shown to enhance cardiovascular endurance, while aquatic resistance training improves muscular strength and endurance (26). Additionally, in a comparative study between high-intensity functional training and running, high-intensity functional training demonstrated significant benefits in muscular fitness, particularly in horizontal jump performance and sit-ups (27). Similarly, another comparative study between high-intensity interval resistance training and running found that high-intensity interval resistance training resulted in superior cardiovascular and muscular fitness (sit-ups) (28), highlighting the influence of exercise intensity and training modality on physical fitness adaptations. A meta-analysis on the effects of yoga practice reported moderate positive effects on muscle strength, balance, flexibility, and lower-body mobility. However, no significant effects were observed on cardiorespiratory endurance or upper-body flexibility (29). In the present study, the control group was not given specific instructions regarding exercise intensity or type, which may have influenced the observed physical fitness outcomes.

The Bajiquan program in this experiment focused on fundamental principles and coordinated movements under the guidance of a coach. In addition to improvements in functional fitness, the program also showed beneficial effects on body composition including reductions in body weight, BMI, and BFP. From the perspective of exercise therapy and physical activity, martial arts can be applied to individuals of all ages for promoting health (2). Physical activity plays a vital role in maintaining physical and mental health. Lack of physical activity not only increases the risk of noncommunicable diseases (NCDs) but also significantly elevates morbidity and mortality rates among individuals affected by these diseases. Engaging in physical activity dose-dependently can reduce the risk of NCDs, such as cardiovascular diseases, type 2 diabetes, and cancer (30). Martial arts remain a niche activity compared to other exercise categories. In a prior survey conducted among Polish participants, the primary motivation for engaging in martial arts was “pleasure”, followed by “maintaining health and physical fitness” and “overall health”, which, in the present study, may be categorized as “personal benefits”. In addition to educational promotion, the availability of facilities and promotional platforms also plays a significant role in participation in martial arts activities (31). This study also conducted a dimensional analysis of health behaviors related to martial arts physical activities, which can be applied in the future to identify facilitating and inhibiting factors across various populations, age groups, educational levels, and cultural backgrounds, thereby highlighting the significance of behavioral engagement.

Moreover, this study showed the benefits of physical activity, highlighting significant improvements in personal fitness, such as physical ability and explosive strength. Martial arts research has revealed the social and psychological benefits and barriers associated with these practices. For instance, judo-related studies have indicated that adult participation in intervention programs can yield psychosocial benefits (e.g., enhanced self-confidence) and physiological improvements (e.g., increased physical fitness) (32). Similarly, Taekwondo interventions have demonstrated notable personal development and self-acceptance among adolescents including increased confidence, happiness, mental satisfaction, improved cognitive abilities, and enhanced focus. Social benefits include fostering positive social relationships, respect for others, kindness, discipline, and patience (33, 34). For the elderly population, Tai Chi interventions provide positive psychological effects, such as increased confidence, self-esteem, and well-being, while alleviating stress and depressive symptoms and improving emotional states (35). Social benefits for this demographic group included expanded social networks and reduced feelings of loneliness (36). Additionally, existing studies have highlighted the role of psychological factors as moderators influencing behavioral changes during activity interventions. Negative attitudes may act as barriers to behavior, whereas factors such as insufficient social support, ineffective teaching methods, and the complexity of movements during interventions can also hinder behavioral engagement (37, 38). In a previous systematic review concerning adherence to lifestyle behaviors including diet and physical activity, three levels of facilitators and barriers were identified as follows: individual (encompassing attitudes, health concerns, and physical changes), environmental (including social support, social accountability, and community factors), and intervention. Lifestyle interventions that promote self-regulatory skills, provide opportunities for social engagement, and allow for the personalization of goals may enhance adherence to desired behaviors (37). However, a systematic review indicated that the multifaceted nature of martial arts may lead to diverse, and sometimes even conflicting, effects on mental health. This highlights martial arts research requires extensive theoretical and practical advancements to better understand its impact on mental health (39).

## 5 Limitation

Regarding the study's experimental limitations, variations in caloric expenditure due to exercise intervention may have affected body composition and physical activity levels. Furthermore, as Bajiquan remains a niche martial art unfamiliar to the public, participants' engagement and training emphasis could have influenced its effects on physical fitness. Given the limited empirical research on Bajiquan, this study aimed to establish a structured training program under the guidance of martial arts instructors and explore its potential health benefits. Future research should include a larger sample size to further investigate its effects following the broader promotion of Bajiquan. Additionally, heart rate monitoring and comparisons with other martial arts could provide deeper insights into the exercise intensity and health benefits associated with Bajiquan practice.

## 6 Conclusion

The Bajiquan training program may serve as a viable alternative physical activity for enhancing physical fitness and supporting weight management as part of health promotion efforts. Additionally, martial arts could influence practitioners' physical fitness, social interactions, psychological barriers, and overall attitude toward exercise. Through the established Martial Arts Exercise Perceived Benefits Questionnaire, this study also provides valuable insights into the unique personal and psychological benefits associated with this martial art.

## Data Availability

The raw data supporting the conclusions of this article will be made available by the authors, without undue reservation.
